# Advances in the study of TIM3 in myelodysplastic syndrome

**DOI:** 10.3389/fimmu.2025.1647401

**Published:** 2025-08-19

**Authors:** Xinyu Guo, Shunjie Yu, Jinglian Tao, Yingshuai Wang, Zonghong Shao, Rong Fu, Lijuan Li

**Affiliations:** ^1^ Department of Hematology, Tianjin Medical University General Hospital, Tianjin, China; ^2^ Department of Hematology, Peking University People’s Hospital, Beijing, China; ^3^ Center for Molecular Medicine, University of Georgia, Athens, GA, United States; ^4^ Department of Hematology, The Affiliated Hospital of Qingdao University, Qingdao, Shandong, China

**Keywords:** T-cell immunoglobulin mucin 3 (TIM-3), myelodysplastic syndrome (MDS), immune checkpoints, immune escape, targeted therapy

## Abstract

Myelodysplastic syndromes (MDS) are heterogeneous myeloid clonal disorders derived from hematopoietic stem cells. The incidence of MDS (1.51/100,000 in China, 4-5/100,000 in Europe and America) is higher than any subtype of leukemia. In recent years, the imbalance of immune regulation and tumor microenvironmental disorders have received increasing attention in the pathogenesis of MDS. T-cell immunoglobulin and mucin-domain containing protein 3 (TIM-3) is an important inhibitory immune checkpoint molecule, widely expressed in T cells, NK cells, and dendritic cells, monocytes/macrophages and other immune cells. Numerous studies have confirmed that TIM-3 is aberrantly expressed in a variety of solid and hematologic tumors and plays an important role in regulating tumor escape and immune depletion. In this paper, we focus on reviewing the relevant studies of TIM-3 in MDS and summarize the findings of our team in this field. We also discuss the potential application of TIM-3 in the diagnosis and treatment of MDS in conjunction with the latest clinical trials. Blocking TIM-3 has both ‘tumor cell-targeted inhibition’ and ‘immune function remodeling’ dual roles in MDS disease progression, which provides new therapeutic strategies and hope for MDS patients.

## 1 Introduction

Myelodysplastic syndromes (MDS) can present with ineffective hematopoiesis, peripheral blood cytopenia, abnormal bone marrow cell development, and a high risk of transformation into Acute Myeloid Leukemia (AML). Currently, there is no effective, safe, and easily accessible treatment for this disease.

In recent years, with the rise of immunotherapy, the use of immune checkpoint inhibitors in tumors has gradually become a hot research topic. It has been found that these molecules are expressed on immune cells and inhibit their activation, which ultimately leads to immune escape of tumor cells and accelerates tumor metastasis and spread. And immune checkpoint inhibitors can block the immune escape of tumor cells and restore the body’s immune recognition and killing of tumors. Inhibitors of immune checkpoint molecules such as PD-1/PD-L1 and CTLA-4 have achieved significant efficacy in a variety of solid tumors and have gradually expanded into the field of hematologic malignancies (1).

The pathogenesis of MDS has not been fully clarified, and the more accepted explanations are: molecular genetic variation of primitive hematopoietic stem cells, proliferation of abnormal precursor cells (2); imbalance of the body’s immunosurveillance system, abnormal bone marrow microenvironment, and disorders of the immune microenvironment. The bone marrow microenvironment is mainly composed of cellular components (immune cells, vascular endothelial cells, osteoblasts and mesenchymal stromal cells, etc.) and bone marrow ecological niche (3). Under the regulation of these immune cells, e.g., MDSC cells and malignant clonal hematopoietic cells in the bone marrow of MDS patients are increased in number and secrete immunosuppressive factors, chemokines, and growth factors to reduce the proliferation of effector T cells and NK cells, the increased number of Treg cells leads to immunosuppression, and the aberrant activation of inflammatory signaling pathways by MSCs drives the development of MDS, etc., which suppresses normal immune responses and causes an Bone marrow inflammatory microenvironment, leading to immune escape of malignant clonal cells, impaired clearance, and ultimately promoting the occurrence and development of MDS/AML (4) (5). Bone marrow microenvironment and immunoinflammatory disorders as one of the key pathogenesis of MDS, so immunosuppressants may become an alternative treatment to demethylating drugs, however, there are still some patients who are insensitive to PD-1/PD-L1 or CTLA-4 monoclonal antibody, which requires us to continue to explore more potential targets in diseases such as MDS.T-cell immunoglobulin mucin 3 (TIM-3) is precisely in this context as a novel immune checkpoint that has received much attention.

TIM-3 is expressed in a variety of immune cells and tumor cells, and regulates immune responses and inflammatory pathways through interactions with its ligands (e.g. Gal-9, HMGB1, CEACAM1 and PtdSer). Numerous studies have shown that high TIM-3 expression is associated with poor prognosis in solid tumors and hematologic malignancies, and is closely linked to processes such as maintenance of tumor stem cells, remodeling of the tumor microenvironment, and immune depletion (6–8). In MDS, the mechanism of action and clinical value of TIM-3 is emerging. In this paper, we will comprehensively review the expression and function of TIM-3 in MDS, explore its immunoregulatory mechanism in the process of disease onset, development and transformation, and describe the current application value of TIM-3 in MDS treatment.

## 2 Structure and biological function of TIM-3

### 2.1 Overview of the TIM family

Members of the TIM family (T cell immunoglobulin and mucin domain family) include TIM-1 to TIM-8, of which TIM-1, TIM-3, and TIM-4 have been clearly identified and well-studied in humans (9). TIM-3 consists of three structural domains: the extracellular region containing the immunoglobulin variable domain (IgV), mucin region, and stalk region, while the transmembrane and intracellular regions are enriched with tyrosine residues for mediating the activation or inhibition of downstream signaling pathways (10). TIM-3 was initially identified in CD4+ helper T cells (Th1 cells) and CD8+ cytotoxic T lymphocytes (CTLs) and was regarded as a negative regulatory receptor. With further research, TIM-3 has also been widely demonstrated in innate immune cells such as dendritic cells, NK cells, monocytes/macrophages, mast cells, etc., and plays a key role in a variety of tumor and inflammatory environments (6–11).

### 2.2 Main ligands and signaling pathways

TIM-3/HMGB1 pathway: which can attenuate the innate immune activation by blocking dendritic cells from recognizing the nucleic acids originating from tumors, and which in turn suppresses tumor immune surveillance (15, 16); TIM-3/PtdSer pathway: helps to clear apoptotic cells and inhibit immune hyperactivation under normal conditions; in tumor or chronic inflammatory environments, it may be exploited by tumor cells to evade immunity (17).

**Figure 1:**
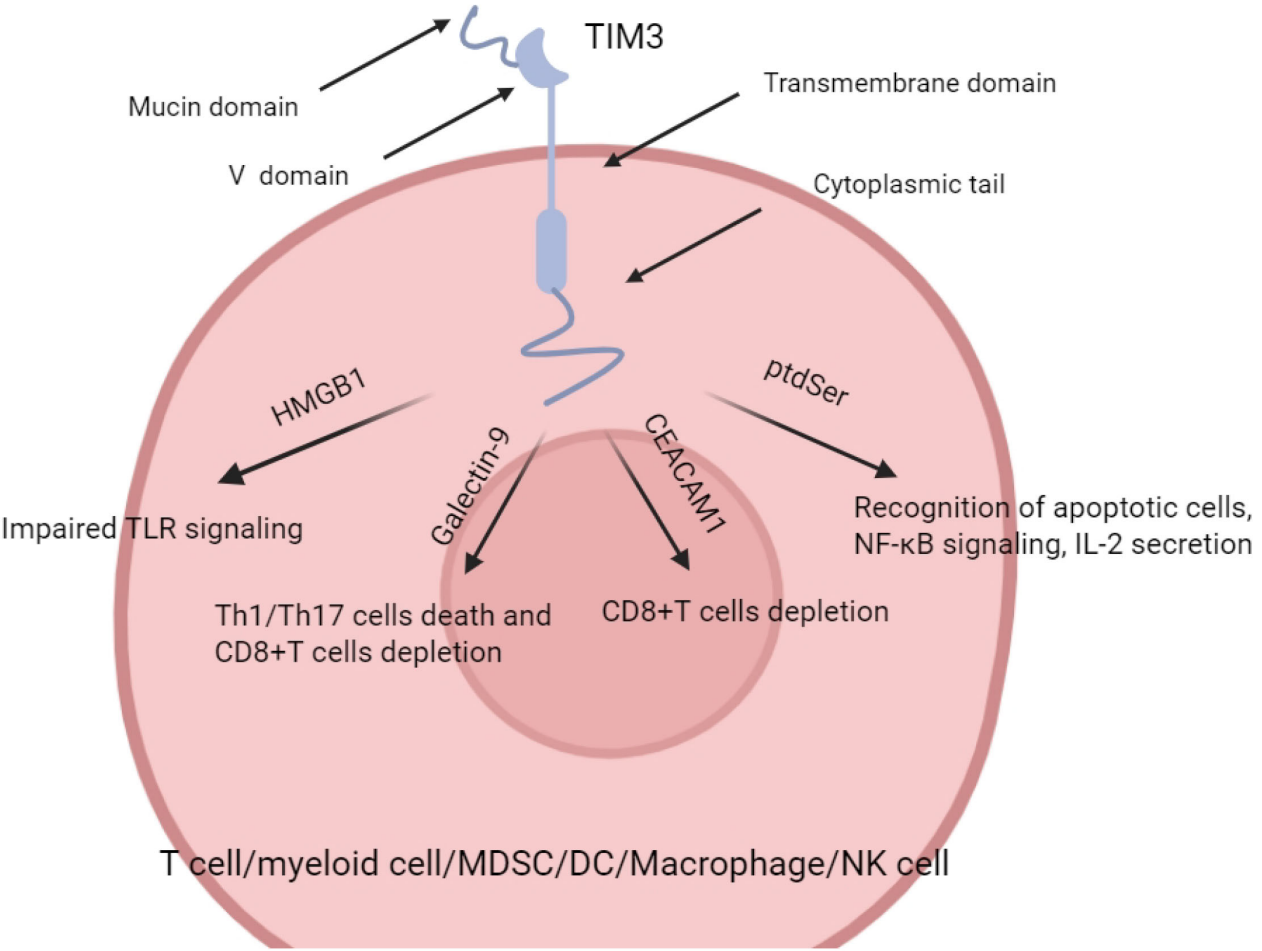
Models for TIm3–ligand (Gal-9 and CEACAM1) interactions. (Cited in: Nat Rev Immunol. 2020 Mar;20(3):173-185).

The identified TIM-3 ligands include galactose lectin-9 (Gal-9), carcinoembryonic antigen-associated cell adhesion molecule 1 (CEACAM1), high mobility group protein 1 (HMGB1) and phosphatidylserine (PtdSer) (12–14). Binding of different ligands to TIM-3 triggers multiple downstream signaling pathways and is involved in the regulation of T cell tolerance, immune depletion, and antigen presentation by dendritic cells. For example: TIM-3/Gal-9 pathway: Binding to Gal-9 on the surface of T cells inhibits IFN-γ secretion and induces apoptosis in Th1 and Th17 cells (11) (15, 16); in the tumor microenvironment, this pathway plays an important role on myeloid-derived suppressor cells (MDSC) and depleted CD8+ T cells (17); TIM-3/CEACAM1 pathway: Our team found that TIM-3 interaction with CEACAM1 not only affects T cell tolerance, but also correlates with the NF-κB/NLRP3/Caspase-1 inflammatory axis, which is involved in inflammation and immune escape in the tumor microenvironment (18); TIM-3/HMGB1 pathway: It can impair innate immune activation by blocking the recognition of tumor-derived nucleic acids by dendritic cells, which in turn suppresses tumor immunosurveillance (19, 20); TIM-3/PtdSer pathway: It helps to clear apoptotic cells and inhibit immune hyperactivation under normal conditions; and may be exploited by tumor cells to evade immunity in tumor or chronic inflammatory environments (21) (**Figure 1**).

Currently, the TIM3-related signaling pathways in AML/MDS are mainly TIM-3/Gal-9 and TIM-3/CEACAM1, while the TIM3/HMBG1 and TIM-3/PtdSer pathways have been less well studied in AML and have not been studied in MDS.

## 3 Role of TIM-3 in hematologic malignancies

### 3.1 TIM-3 in AML and leukemia stem cells

In acute myeloid leukemia (AML), TIM-3 is an important surface marker on leukemia stem cells (LSC). Studies have shown that TIM-3 is highly expressed on LSC but not or lowly expressed on normal hematopoietic stem cells (HSC), and that blocking TIM-3 not only slows down the leukemic progression in AML mice, but also reduces the number of leukemic stem cells transplanted into mice (22). Our team compared the TIM3 expression levels of HSCs in MDS patients, AML patients, and healthy volunteers, and found that the TIM3 expression levels of HSCs in high-risk MDS and AML patients were abnormally high, and TIM3+ HSCs exhibited aberrant differentiation, hyperproliferation, and reduced apoptosis (24). Thereafter, Japanese scholars further suggested that the interaction of TIM-3 with Gal-9 could promote LSC proliferation by activating signals such as NF-κB and β-catenin, and was closely associated with poor prognosis (23).

In addition, Vadim V Sumbayev’s team found that TIM3 interacts with HMGB1 and induces the secretion of VEGF (angiogenic protein vascular endothelial growth factor), which promotes bone marrow angiogenesis, thereby alleviating hypoxic conditions induced by an increased number of LSC cells, which in turn supports the survival and proliferation of LSCs (25). PtdSer is considered to be one of the key one of the “eat-me signals” and promotes the uptake of apoptotic cells. Last year, Fredrik B Thorén et al. performed a genetic screen on the K562 leukemia cancer cell line and found that deletion of the TMEM30A gene leads to the accumulation of PtdSer on the outer side of the cell membrane, which binds to TIM3, which in turn inhibits NK cells leading to immune escape. The results of this phenomenon were consistent across a variety of leukemia cell lines and lymphoma cell lines, and targeted blockade of PtdSer or TIM3 reversed the occurrence of immune escape in TMEM30A-deficient tumor cells (26). In conclusion, the above findings further demonstrate that TIM3 plays an important role in AML pathogenesis and that combining multiple targets (e.g., TIM-3 with other leukemia-associated molecules) to inhibit LSC exhibits stronger anti-leukemic activity than a single target (27).

### 3.2 Progress of TIM-3 in MDS

#### 3.2.1 Abnormal expression of TIM-3 in MDS hematopoietic stem/progenitor cells and osteoblasts

Recent studies have revealed that the immune checkpoint molecule TIM3 is aberrantly expressed in a variety of malignant hematologic diseases.Our team’s TIM-3 assay of bone marrow hematopoietic stem cells (HSC) from MDS patients revealed that TIM-3-positive HSC with aberrant differentiation, hyperproliferation, and reduced apoptosis were strongly associated with higher conversion rates and shorter median survival in patients (24). In addition, in the bone marrow microenvironment of MDS patients, osteoblast activity is significantly reduced and TIM-3 is abnormally highly expressed in osteoblasts, and this high expression may further perturb the balance of the bone marrow ecological niche and promote disease progression (25).

Our team further found that despite the similar morphology of TIM3+ and TIM3- stem cells in MDS patients, TIM3+ stem cells had lower colony-forming ability and more pronounced karyotypic abnormalities, suggesting that they may represent early malignant clones (24). Meanwhile, myeloid-derived suppressor cell (MDSC) cells highly expressed TIM3, CEACAM1, and Gal-9, and inhibited apoptosis of TIM3+ stem cells through the TIM3/Gal-9 and TIM3/CEACAM1 pathways, whereas targeted blockade of the pathways reversed their anti-apoptotic effects (17, 18). We again confirmed by animal models that TIM3+ stem cells, especially in combination with MDSC, showed enhanced expansion capacity *in vivo* but impaired differentiation potential, further supporting their malignant clonal properties and the pro-cancer role of MDSC (24).

The study by Toshio Asayama’s team provides an important addition to the role of TIM3 in MDS progression. They demonstrated again that TIM3 expression on the surface of primitive cells and plasma levels of the TIM3 ligand, galactoselectin-9 (Gal-9), are dynamically elevated with the transformation of MDS to AML and are closely associated with primitive cell proliferation, disease progression, and prognosis (27). In addition, the team demonstrated that the bone marrow microenvironment induced the upregulation of TIM3 and Gal-9 expression, and thus they concluded that the TIM3-Gal-9 signaling axis may contribute to MDS disease progression and transformation to AML.

In conclusion, these findings collectively model the multiple roles of TIM3 in the pathogenesis of MDS, where TIM3 acts as a primitive cell-intrinsic regulator to promote malignant clonal proliferation and disrupts bone marrow microenvironmental homeostasis as well as promotes disease progression and accelerates leukemic transformation in conjunction with the ligand Gal-9. This provides a new rationale for the development of antitumor therapies targeting TIM3.

#### 3.2.2 Role of TIM-3 in the immune microenvironment of MDS

In addition to its pro-proliferative and anti-apoptotic roles in MDS malignant clones, aberrant expression of TIM-3 in immune cells further exacerbates immune escape and disease progression in MDS. Our team analyzed in bone marrow samples from MDS patients by multicolor flow cytometry and found that compared to healthy controls, the proportion of the TIM3+ subpopulation of CD8+ T cells was significantly elevated in MDS patients, but the secretion of granzymes and perforin by this population of cells was decreased, along with the up-regulation of expression of apoptosis-sensitive marker CD95 (Fas), which suggests that there is functional exhaustion of TIM-3+ CD8+ T cells (28). In addition, PD-1 co-expression of TIM3+ CD8+ T cells was significantly elevated compared to controls, suggesting that TIM3 may synergize with other immune checkpoint molecules to jointly mediate T cell dysfunction (29). Subsequently, we have elucidated that TIM3 can regulate the formation of the immunosuppressive microenvironment in MDS through different ligand-dependent pathways: The TIM-3/Gal-9 signaling axis promotes myeloid-derived suppressor cell (MDSC) expansion and induces CD8+ T-cell functional depletion (17); whereas, TIM-3/CEACAM1 interaction, which in turn enhances the immunosuppressive capacity of MDSC, promotes secretion of inhibitory cytokines such as IL-10 and TGF-β, which ultimately exacerbates the bone marrow inflammatory microenvironment (18). Asayama et al. proposed on this basis that the TIM-3/Gal-9 signaling axis and imbalanced bone marrow microenvironment not only contribute to the pathogenesis of MDS, but also accelerates the transition of MDS to secondary AML (sAML) by inducing proliferation of progenitor cells and immune escape and thereby accelerating MDS) transformation (27).

In addition to CD8+ T cells, aberrant expression of TIM3 in the helper T cell (Th) subpopulation also affects immune homeostasis in MDS. Our team found that TIM-3 expression was significantly upregulated in Th1, Th17 and regulatory T cells (Treg) in MDS patients, and, of particular importance, TIM-3+ Treg cells exhibited dysfunction and their TGF-β secretion capacity was reduced (30), suggesting that TIM-3 may weaken the inhibitory capacity of Treg on effector T cells by altering its cytokine profile and while enhancing the overall immunosuppressive microenvironment and playing an important role in immune escape.

Recent studies have also revealed the critical role of TIM3 in intrinsic immune cells. In dendritic cells (DCs), TIM3 maintains the tolerogenic phenotype of DCs by inhibiting NLRP3 inflammatory vesicle activation. Knockout experiments confirmed that TIM3-deficient DC cells significantly enhanced the activation and expansion of CD8+ T cells and stem cell-like T cells (TSCM), and promoted anti-tumor immune responses (31). In addition, the expression of TIM3 in the monocyte-macrophage system also has a dual regulatory role: on the one hand, TIM3+ macrophages exhibit an M2-type polarization tendency, with impaired phagocytosis and antigen-presentation; on the other hand, TIM3 can promote the secretion of immune-suppressive cytokines through the regulation of the NF-κB signaling pathway, which further deteriorates the inflammatory microenvironment of MDS (32), allowing the tumor cells to evade immune surveillance and attack the organism, accelerating tumor progression and immune escape.The immunomodulatory role of TIM3 was further supported by the clinical study of Moiseev et al. who found that the proportion of TIM3-positive NK cells (CD56+TIM3+) was significantly increased in patients with MDS and, together with CD8+TIM3+T cells and CD4+TIM3+T cells, constituted the immune signature of poor prognosis. Multivariate analysis showed that patients with high TIM3-expressing immune cell populations had shorter progression-free survival (PFS) and worse prognosis (33).

In summary, these findings point to the conclusion that the aberrant expression of TIM-3 in MDS progenitor cells and immune cells builds a complex regulatory network: TIM3 directly inhibits the anti-tumor activity of T/NK cells, promotes the immunosuppressive function of MDSC and M2-type macrophages, and alters the immune-regulatory properties of DC cells and Treg cells. These effects form a “tumor-immune microenvironment” positive feedback loop that drives disease progression. Therefore, therapeutic strategies targeting TIM-3 (e.g., TIM3 monoclonal antibody or combined PD-1/CTLA-4 blockade) may not only directly inhibit tumor growth, but also reshape the immune microenvironment by lifting the suppression of DC cells by TIM3, enhancing the cross-presentation capacity of tumors, and activating CTL cells; reprogramming the polarization of macrophages to enhance phagocytosis; and restoring the virulence and proliferative capacity of NK/T cells, thus reversing the suppression of the immune microenvironment, and improving the immunoregulatory properties of DC cells and Treg cells. Reversing the suppressed state of immune microenvironment, improving new direction for MDS immunotherapy. However, due to the great heterogeneity of MDS, the degree of immunosuppression and microenvironment of different patients are also different, and there may be differences in the efficacy of TIM3 inhibitors after application.

### 3.3 Significance of TIM-3 in other hematologic tumors

TIM3 also showed high expression levels in other hematological malignancies, and our team found that high expression of TIM-3 was present on myeloma cells of multiple myeloma (MM) patients and correlated with disease progression, and was also found to be closely related to the activation of the NF-κB signaling pathway; knockdown of TIM-3 significantly inhibited cell proliferation and induced apoptosis, and bortezomib had a synergistic NF-κB pathway inhibition, suggesting that TIM-3 could be a potential future therapeutic target for MM (34). It has been found that the expression of TOX, TOX2 protein and TIM3 is elevated in T-cell acute lymphoblastic leukemia (T-ALL), and that TOX and TOX2 proteins can directly induce the transcription and expression of TIM3, preventing apoptosis, whereas targeting TXO or TIM3 slows down the growth of tumors (35). A study found that with the disease progression of B-cell acute lymphoblastic leukemia (B-ALL), the expression of TIM-3 in T cells and its ligand galectin-9 were significantly upregulated in both primitive cells and MSCs, and the upregulation of the expression of TIM-3 and galectin-9 was negatively correlated with the disease prognosis, and the study also demonstrated that CAR19-TIM3- Fc T cells could promote the expansion of transduced and memory T cells *in vivo* and improve the antileukemic efficacy and durability of CAR19 T cells in B-ALL (36), however, the results of another study on TIM3 in B-ALL were contradictory, they also found that the expression of TIM3 was elevated in B-ALL CD34+CD19+B primitive cells, but TIM3+B primitive cell transplanted mice showed no significant difference in EFS and OS from TIM-B primitive cell transplanted mice (37). There is increasing evidence that TIM3 expression is elevated in CML stem cells, CD4+ and CD8+ T cells in both primary and relapsed patients with chronic myeloid leukemia (CML), inducing T-cell depletion, and that blocking TIM3 may improve the immune response generated by the discontinuation of TKI inhibitors and concurrently target leukemic stem cells, preventing the disease from relapsing (38, 39). In addition, the role of TIM-3 in chronic lymphocytic leukemia (CLL) and certain lymphomas has been gradually gaining attention, but more mechanistic and clinical studies are needed to clarify the specific role played by TIM3 and to confirm the clinical value of TIM3.

## 4 Novel therapeutic strategies and clinical progress related to TIM-3

### 4.1 Monoclonal antibody monotherapy and combined multi-target blockade programs

Based on the multiple mechanisms of TIM-3’s role in tumor and immunity, a variety of monoclonal antibodies against TIM-3 have entered the preclinical and clinical research stage in the last decade, including blockade of TIM-3 alone and combined blockade with other immune checkpoints, such as PD-1/PD-L1, CTLA-4, LAG-3, TIGIT, etc. (40, 41). For myeloid tumors, the significance of TIM-3-targeted therapy is even more prominent: not only may it directly inhibit the proliferation of primitive/stem cells, but also partially restore the immune depletion of T cells or NK cells.

### 4.2 TIM-3 blockade in combination with demethylating drugs

In the treatment of MDS and AML, demethylating agents (HMAs) such as azacitidine (AZA) and decitabine (DAC) are widely used, but drug resistance and relapse still occur in most patients. Some investigators have tried to combine TIM-3 monoclonal antibody with HMA and found that it can enhance inhibition of tumor cells and improve the immune microenvironment. Several clinical trials are currently evaluating the efficacy of such combination regimens in MDS and AML.

Several clinical trials have been conducted in combination with demethylating agents. Sabatolimab (MBG453) is a humanized IgG4 anti-TIM-3 monoclonal antibody that specifically binds to TIM-3 and blocks its binding to ligands. STIMULUS-MDS1 (NCT03066648): enrolled patients with high/very high-risk MDS versus newly diagnosed patients with primary AML. The study showed an overall favorable safety profile for combination therapy in both the MDS and AML populations, with higher remission rates (both complete and partial) in the MDS group compared to the AML group, and some clinical benefit in some patients with adverse risk gene mutations (e.g., TP53). In another phase II trial (NCT04150029), 18 patients with AML were enrolled and given a three-drug combination of Sabatolimab + Venetoclax + Azacytidine, which was shown to be comparable in safety and tolerability to the two-drug combination of Venetoclax + Azacytidine. The preliminary results of these trials provide important evidence for the use of TIM-3 monoclonal antibody in MDS: in combination with demethylating drugs, it can enhance the response of high-risk MDS patients to a certain extent and is well tolerated, bringing new therapeutic hope for MDS patients.

### 4.3 TIM-3-CAR-T and bispecific CAR-T/CAR-NK

In the field of cell therapy, chimeric antigen receptor T cell (CAR-T) technology has been successfully applied to a variety of B-cell tumors. In recent years, TIM-3 has also been studied as a target for AML or MDS cells and introduced into CAR-T cells to selectively kill leukemia cells with high TIM-3 expression (42). In addition, some teams have also explored bispecific CAR-T, such as targeting both TIM-3 and CD13, which demonstrated higher tumor clearance and relatively controllable toxicity to normal hematopoietic stem cells in AML mouse models (43). To further minimize the possible adverse effects of CAR-T such as severe cytokine release syndrome (CRS) and neurotoxicity, some investigators are trying to introduce CAR into NK cells (CAR-NK) (44). Preliminary results show that TIM-3-CAR-NK exhibits better anti-tumor activity in both *in vitro* and ex vivo experiments. Although such studies are still in the early exploratory stage, they offer new possibilities for personalized cell therapy for MDS and AML.

## 5 Latest clinical trial progress and challenges

With more clinical trials, the mechanism of action and efficacy of TIM-3 inhibitors in hematologic tumors have been further confirmed. However, the following challenges need to be noted:

### 5.1 Combined blockade with other immune checkpoints

The current clinical results regarding TIM3 monoclonal antibody monotherapy for MDS disease are not satisfactory, and TIM3 inhibitors need to be co-applied with other target drugs to achieve the expected results. According to the results of clinical trials of TIM3 in MDS/AML/CMML (NCT04878432,NCT04812548,NCT03066648,NCT03946670), the combination of TIM3 inhibitors with demethylating drugs (decitabine or azacitidine) and/or small-molecule targeted drugs or immune checkpoint inhibitors is better than monotherapy. better than single-agent application, partly because of biased results due to too few recruits, and partly because checkpoint molecules such as TIM-3, PD-1, CTLA-4, LAG-3, and TIGIT may play different roles at different stages and in different cell types, or there may be mutual compensation. It is sometimes difficult to achieve sustained clinical remission by blocking a pathway alone, and multi-agent combinations are not only efficacious but also safer for patients who are not suitable for intensive chemotherapy or after stem cell transplantation. However, more clinical studies are needed to overcome the clinical challenge of optimizing the timing, dosage and strategy of combination therapy, as well as assessing patient resistance and tolerability.

### 5.2 Evaluation of efficacy in patients with adverse risk gene mutations

Mutations such as TP53 are prevalent in high-risk MDS/AML and the prognosis is usually poor. Whether the trial data suggest that TIM-3 blockade may also have some efficacy in such patients requires further large-scale validation.

### 5.3 Immune-related toxicities and drug resistance

Similar to other immunotherapies, TIM-3 blockade may bring autoimmune or inflammatory side effects, such as over-immune activation and myelosuppression, etc. Moreover, new immune escape pathways may emerge in the tumor cells and the microenvironment, which may lead to secondary drug resistance.

### 5.4 Compared with other immune checkpoint-targeted drugs

there have been hundreds of clinical trials of immune checkpoint inhibitors for MDS currently under investigation, such as TIM3, PD-1/PD-L1, CD47, CTLA-4, Clever-1 inhibitors, etc. However, none of the clinical effects of single-agent therapy are satisfactory, and the core strategy of treatment is still combination of demethylating drugs, and the combination of drugs in the primary treatment of higher-risk MDS patients with ORR up to 60-80% (NCT03066648, NCT04623216, NCT03248479, NCT05428969). In comparison, TIM3 inhibitors have a slightly weaker ORR than PD-1 and Clever-1 inhibitors, but have a stronger overall safety profile, with no typical irAE occurring at present, and may be more suitable for MDS patients intolerant of PD-1 toxicity.TIM3 is uniquely advantageous in that it can target both T-cells and myeloid tumor cells, making it more suitable for patients with a highly suppressed immune microenvironment (e.g., high Treg infiltration).

In recent years, the development of tumor immunotherapy has provided new therapeutic ideas for malignant hematological diseases such as MDS, etc. TIM-3, as an important immune checkpoint molecule, is often highly expressed in myeloid and lymphoid tumor cells on the one hand, which promotes malignant proliferation and immune escape, and on the other hand, it can also be expressed on a wide range of immune cells (e.g., T cells, NK cells, DC cells, macrophages, etc.), which affects immune effects and inflammatory microenvironment. For MDS, the mechanism of action of TIM-3-targeted therapy may combine the advantages of “tumor cell inhibition” and “immune activation”, but more large-scale phase III trials are needed to validate the survival benefit, and it may become an alternative option for PD-1-resistant or highly immunosuppressive MDS in the future. In the future, it may become an alternative choice for PD-1-resistant or highly immunosuppressive MDS.

## 6 Discussion

TIM-3, as an emerging inhibitory immune checkpoint molecule, plays an important role in the pathogenesis of myeloid malignant tumors, especially MDS and AML. At the tumor cell level, high expression of TIM-3 promotes the proliferation and anti-apoptosis of primitive cells and LSC; at the level of the immune environment, TIM-3 induces the depletion of T cells and NK cells, and regulates inhibitory cell populations, such as MDSCs, dendritic cells, and macrophages, to fuel immune escape; at the level of the myeloid ecological niche, TIM-3 may be associated with the dysfunction of osteoclasts and stromal cells, and TIM-3 may be associated with osteoblast and stromal cell dysfunction at the bone marrow ecological level, and influence the progression of MDS through inflammatory signaling axes (e.g., NF-κB/NLRP3).

Immunotherapy, with its unique mechanism of “remodeling the body’s immune system to recognize and kill tumors”, is bringing new therapeutic hope for a variety of malignant hematological diseases. Monoclonal antibodies against TIM-3 (e.g. Sabatolimab) and their combination with other immune checkpoint inhibitors or HMA, BCL-2 inhibitors, etc. have achieved certain results in MDS/AML clinical trials, and some patients have achieved long-lasting remission, while cellular therapeutic strategies, such as CAR-T/CAR-NK, have provided a new way of thinking for refractory relapse cases. However, there are still many challenges: for example, the difference in efficacy of TIM3 inhibitors in different disease stages, and how to combine with other checkpoint inhibitors or chemotherapeutic agents in order to obtain the optimal synergistic effect.

Based on the current challenges, future MDS-related studies could further explore the association between TIM3 ligand expression and the degree of myeloid cell infiltration and efficacy. Meanwhile, we should pay more attention to patients with refractory relapsed or drug-resistant MDS, clarify the mechanism of drug resistance and the role of immune microenvironmental disorders, deepen the understanding of the pathogenesis and drug resistance mechanisms, and increase the number of clinical trials of TIM3 multidrug combination therapy for MDS, so as to provide more clinical basis for TIM3 combined with PD-1/PD-L1, Clever-1 and other inhibitors.TIM3 inhibitors in clinical application should pay attention to the combination of molecular typing, immune microenvironment inhibition stratification and dynamic monitoring, so as to develop a more precise and individualized immunotherapy program.

In summary, the study of TIM-3 provides new possibilities for the pathogenesis and clinical treatment of MDS. With the accumulation of evidence from more large-scale clinical trials, TIM-3 is expected to become a key molecule in the precision treatment and immunotherapy of MDS, and may play an indispensable role in improving the prognosis of MDS patients in the future.
